# Evolutionary change in physiological phenotypes along the human lineage

**DOI:** 10.1093/emph/eow026

**Published:** 2016-09-11

**Authors:** Alexander Q. Vining, Charles L. Nunn

**Affiliations:** 1 Department of Evolutionary Anthropology, Duke University, Durham, NC, USA; 2 Duke Global Health Institute, Duke University, Durham, NC, USA; 3 Triangle Center for Evolutionary Medicine, Durham, NC, USA

**Keywords:** human physiology, comparative analysis, primates, human evolution

## Abstract

**Background and Objectives:** Research in evolutionary medicine provides many examples of how evolution has shaped human susceptibility to disease. Traits undergoing rapid evolutionary change may result in associated costs or reduce the energy available to other traits. We hypothesize that humans have experienced more such changes than other primates as a result of major evolutionary change along the human lineage. We investigated 41 physiological traits across 50 primate species to identify traits that have undergone marked evolutionary change along the human lineage.

**Methodology:** We analysed the data using two Bayesian phylogenetic comparative methods. One approach models trait covariation in non-human primates and predicts human phenotypes to identify whether humans are evolutionary outliers. The other approach models adaptive shifts under an Ornstein-Uhlenbeck model of evolution to assess whether inferred shifts are more common on the human branch than on other primate lineages.

**Results:** We identified four traits with strong evidence for an evolutionary increase on the human lineage (amylase, haematocrit, phosphorus and monocytes) and one trait with strong evidence for decrease (neutrophilic bands). Humans exhibited more cases of distinct evolutionary change than other primates.

**Conclusions and Implications:** Human physiology has undergone increased evolutionary change compared to other primates. Long distance running may have contributed to increases in haematocrit and mean corpuscular haemoglobin concentration, while dietary changes are likely related to increases in amylase. In accordance with the pathogen load hypothesis, human monocyte levels were increased, but many other immune-related measures were not. Determining the mechanisms underlying conspicuous evolutionary change in these traits may provide new insights into human disease.

## INTRODUCTION

Physiological mechanisms relevant to human health and disease have been studied in depth, yet the evolution of these processes is less well understood. Investigating the evolution of physiological processes may shed light on why they fail or become unhealthy. For example, fitness trade-offs may be more likely to occur when rapid evolution in one trait generates correlated costly effects on other traits or bodily functions, especially when coupled with genetics, developmental or morphological constraints that inhibit the ability of natural selection to respond to these costs (1). Indeed, it is well established that many genes associated with disease have pleiotropic effects that could generate such tradeoffs, including for traits involved with physiology (2).

One approach to investigate the evolution of human physiology involves comparing humans to our closest evolutionary relatives, the primates. This comparative approach is central to investigating the evolution of phenotypes, and physiology has long been a focus of comparative research. For example, Huey and Bennett (3) used comparative approaches to investigate temperature and running performance in lizards, while Kleiber’s (4) studies of metabolic rate in relation into body mass have had broad impacts on a wide range of fields, such as the metabolic theory of ecology (5). Similarly, using data on physiological reference values for primates, Lindenfors *et al.* (6) investigated sex differences in aerobic capacity, finding that males have higher haematocrit (red blood cells per blood volume) than females.

Most phylogenetic comparative methods test adaptive hypotheses or map the origins of traits onto a phylogeny (7–9). Comparative studies can also be used to assess whether a species has undergone exceptional or rapid evolution (10–12). For example, Organ *et al.* (13) investigated dental morphology and behaviour along the human lineage. They showed that the origins of food processing (such as cooking) have led to shorter durations of feeding per day. They further used dental morphology to pinpoint the timing of the origins of food processing in fossil hominins by reconstructing the molar area using phylogenetic and statistical modelling. Similarly, Samson and Nunn (14) used a phylogenetic comparative approach to investigate human sleep, relative to other primates. They discovered that human sleep is substantially shorter than predicted based on phylogeny and patterns of trait covariation in other primates. These and similar approaches might be relevant for understanding the generation of tradeoffs relevant to disease. For example, reduced sleep could enable more time for learning or building social alliances that lead to higher fitness, but could have costly health or cognitive effects later in life.

Here, we investigate physiological traits that have undergone exceptional evolution along the human lineage. By focusing on 41 physiological ‘reference values’—which are widely used in medical diagnostics due to their relationships with disease states—we aim to identify whether and how human disease-relevant physiological traits differ from other primates. We used two different methods to investigate evolution along the human lineage that make different assumptions and capture different aspects of the evolutionary process. The first method—phylogenetic prediction—uses generalized linear models to predict trait values in humans, and then quantifies the degree to which humans depart from predictions (12,13). When humans differ from expectations, they are identified as ‘evolutionary outliers’. The second method—evolutionary modelling—infers adaptive regimes across the primate phylogeny under an Ornstein-Uhlenbeck (OU) model of evolution. These adaptive regimes enforce a central tendency on trait values, with a trait optimum, variance and strength of selection that can vary across the branches of a phylogeny. With the evolutionary modelling approach, we specifically investigated whether trait optima have shifted on the branch leading to humans. Taken together, these two analyses identify traits that have experienced novel selective pressures on the human lineage, relative to other primates.

The specific functions of the physiological traits that we investigated (Table 1) are understood in the context of health and disease. For example, low red blood cell counts (anaemia) can cause fatigue and dizziness, while high cholesterol is associated with coronary heart disease. However, the adaptive significance of interspecific variation in these traits is less well understood. Although extensive effort has been made to understand the healthy ranges of physiological traits for humans and other animals, virtually no research has considered why these traits vary across species. Thus, for most of the physiological reference values, *a priori* predictions for human differences are not possible.

**Table 1.** Traits Analysed.

Trait	Health associations
Alanine aminotransferase	liver injury
Alkaline phosphatase	liver and bone disease
Amylase	pancreatitis
Aspartate aminotransferase	liver disease
Basophils^a^	cancer (low), vascular disease (high)
Bicarbonate	electrolyte and acid-base imbalance
Blood urea nitrogen	liver failure (low), kidney disease (high)
Body temperature	thermoregulation
Calcium	bone, liver, kidney disease
Carbon dioxide	kidney and lung malfunction
Chloride	Addison disease (low), metabolic and renal tubular acidosis (high)
Cholesterol	heart disease
Creatine phosphokinase	injury or stress to muscle, heart, or brain
Creatinine	kidney function
Eosinophils^a^	Addison disease, cancer
Gamma glutamyltransferase	liver disease
Glucose	Diabetes
Haematocrit^b^	anemia, leukaemia
Haemoglobin^b^	anemia, chronic kidney disease
Indirect bilirubin	Jaundice
Iron	Anemia
Lactate dehydrogenase	tissue damage
Lipase	pancreatitis
Lymphocytes^a^	HIV, leukaemia (low), hepatitis (high)
Magnesium	loss of kidney or adrenal gland function
MCH^b^	iron deficiency (low), folate deficiency, liver disease (high)
MCHC^b^	iron deficiency (low), hereditary spherocytosis (high)
MCV^b^	iron deficiency (low), folate deficiency, liver disease (high)
Monocytes^a^	leukaemia, tuberculosis
Neutrophilic bands^a^	immune response
Osmolarity	fluid balance
Phosphorus	hyperthyroidism (low), kidney, liver failure (high)
Platelet count	bone marrow diseases, cancer
Potassium	Conn syndrome (low), kidney disease, infection (high)
Red blood cell count^b^	nutritional deficiency, bone marrow damage (low), lung disease (high)
Segmented neutrophils^a^	eclampsia
Sodium	problems with adrenal glands
Total bilirubin	Jaundice
Triglyceride	heart disease
Uric acid	diabetes, leukaemia, renal failure (high)
White blood cell count^a^	bone marrow disorders, autoimmune conditions (low), inflammation, leukaemia, alergies (high)

^a^Traits predicted to increase as a result of increased pathogen load.

^b^Traits predicted to increase as a result of long distance running.

A list of all traits included in the analysis with potential health effects when that trait fall outside normal human ranges

For a subset of traits, however, we can formulate specific hypotheses and predictions to test. We focus on two hypotheses. First, under the hypothesis that long-distance running resulted in many human-specific adaptations (15), we predict increases in blood parameters associated with more efficient oxygen transport. Specifically, we predicted increases in total haemoglobin and/or its concentration, which could be reflected in increased red blood cell counts, haemoglobin, haematocrit, mean corpuscular haemoglobin (MCH, average mass of haemoglobin per red blood cell), mean corpuscular haemoglobin concentration (MCHC, concentration of haemoglobin in a given volume of packed red blood cells), and mean corpuscular volume (MCV, mean volume of red blood cells).

The second hypothesis concerns the importance of pathogens in human evolution, based on the acquisition of parasites and pathogens from domesticated animals (16) and evidence that the pathogen environment has been a major selective pressure (17). This hypothesis predicts higher than expected values for overall white blood cell counts (12) and specific white blood cell types, such as eosinophils, which are important for fighting macroparasites (18), and segmented neutrophils, an important defence against bacterial pathogens (19). With results from analyses of each of these traits, we then investigated the more general hypothesis that human physiology differs from other primates.

## METHODOLOGY

### Data collection

Data on animal physiology were attained from the International Species Information System (ISIS) (20). ISIS aggregates physiological measurements taken primarily from zoo animals for the purpose of establishing improved veterinary care. From this database, we extracted all records for primates, focusing on average female values to control for differences between sexes that arise through interspecific variation in the degree of sexual selection operating on males. We only included data with values from at least ten animals, and we excluded physiological traits when fewer than ten species of primates had qualifying data. In addition, traits with values that are highly variable over time (e.g. cortisol or progesterone) or had no comparable trait commonly measured in humans (e.g. tocopherol) were also excluded. We included body mass in the analysis because this trait is known to correlate with life history, diet and disease risk (parasite richness), all of which are likely to influence the physiological traits we investigated. In addition, data on primate body mass are widely available, often from the same individuals that provided data on physiological reference values (except for *Callicebus donacophilus, Callicebus moloch, Macaca nigra* and *Miopithicus talapoin*, which were missing from ISIS and thus obtained from Primate Info Net, http://pin.primate.wisc.edu/; accessed 21 October 2014). Human physiological reference values were primarily attained from appendices and tables in Bope and Kellerman (21), using the midpoint of the range provided and again focusing on females when sex-specific values were provided. The exceptions were amylase, osmolarity, uric acid and phosphorous, which were acquired from MedlinePlus (https://www.nlm.nih.gov/medlineplus/encyclopedia.html; accessed 21 October 2014). All data, including body mass, were log_10_ transformed prior to analysis.

### Phylogenetic prediction

Human values for each trait were compared to predictions made by BayesModelS (12), an R implementation (22) of methods similar to those first introduced by Organ *et al.* (23). BayesModelS takes as input a posterior distribution of phylogenies, a regression formula and values for the predictor and response variables for a set of species, excluding species from the tree and dataset without equivalent matching data. BayesModelS then generates a posterior distribution of regression models using reversible-jump Markov Chain Monte Carlo (MCMC). The parameters estimated in each model include which predictor variables to include in the model (a Bayesian model selection procedure), the coefficients for included predictors, and a tree parameter (λ) that scales branch lengths to reflect phylogenetic signal. Specifically, λ scales the internal branches of the phylogeny by a value between 0 and 1. When λ = 0, this generates a star phylogeny (24), which is equivalent to a non-phylogenetic analysis, while increasing values of λ (approaching 1) indicate stronger phylogenetic signal. In each step of the MCMC, parameters may be added or dropped from the model, and parameter values can change. The changes made to each parameter are mediated by a set of predetermined prior distributions. If the changes increase the likelihood of the data, the parameters are updated to the new values. If the changes reduce the likelihood, they are kept with a probability inversely proportional to the magnitude of the reduction in likelihood; otherwise values revert to their previous state before making another set of changes.

Each model in the posterior distribution can be used to generate a distribution of possible values for a ‘target species’ that was not included when estimating model parameters. These distributions incorporate the predictor variables for the target species of interest and the phylogenetic position of that target species, and control for sources of uncertainty in phylogeny and parameter estimation. A single value is drawn from each of these distributions, generating a set of predicted values for the target species. One can compare the measured trait in the target species to this distribution to quantify any difference from predictions. When the actual value is sufficiently far from the predicted distribution (see below), the species is an ‘evolutionary outlier’ relative to other species in the clade; the trait is greater or less than predicted based on phylogeny and trait covariation amongst the species.

For this analysis, the target species was *Homo sapiens*. One hundred primate phylogenies were downloaded from 10kTrees Version 3 (25) and pruned for all species not included in a given analysis of one of the 41 physiological reference values. The actual number of species varied by trait (see Table 2). The MCMC was set to include female body mass of each species as the predictor variable, and a uniform prior was set for the regression coefficient. The prior distribution of λ was also set to be uniform, ranging from 0 to 1. For each trait under investigation, the chain was run for 200100 steps, with the first 100 discarded as burn-in and every 100 steps after that selected for inclusion in the final posterior distribution (‘thin’ rate). This produced posterior probability distributions of 2000 model parameters (and predictions). Before running any statistical tests on these distributions, we checked the likelihood of models across the chain to ensure minimal auto-correlation of models. We also visually inspected the distribution of human predictions for each trait for normalcy using Q-Q plots before calculating the difference between the observed and predicted means, and the probability of obtaining a difference that size or larger. From these probabilities, we identified evolutionary outliers using a significance threshold determined by controlling false discovery rate (26), with false discovery rate set to 10%.

**Table 2.** Summary of posterior distribution of predictions by BayesModelS.

Trait	# of Species	Units (log)	Mean prediction	SD prediction	Human value	Probability	Extreme species
Alanine aminotransferase	50	U/l	1.42	0.77	1.35	0.71	3
Alkaline phosphatase	50	U/l	2.46	0.01	1.97	**0.01**	1
Amylase	35	U/l	0.87	0.00	1.81	**<0.01**	3
Aspartate aminotransferase	50	U/l	1.32	0.57	1.39	0.56	2
Basophils	37	*10^^^9/l	−1.16	0.05	−1.42	0.16	2
Bicarbonate	16	mMol/l	1.41	0.87	1.39	0.71	2
Blood urea nitrogen	50	mMol/l	0.59	0.04	0.78	0.03	2
Body temperature	44	C°	1.56	0.52	1.57	0.55	3
Calcium	50	mMol/l	0.37	0.66	0.38	0.63	4
Carbon dioxide	32	mMol/l	1.39	0.65	1.43	0.31	3
Chloride	48	mMol/l	2.01	0.53	2.00	0.60	2
Cholesterol	49	mMol/l	0.76	0.74	0.72	0.73	4
Creatine phosphokinase	40	U/l	2.42	0.01	2.00	**0.01**	3
Creatinine	50	μMol/l	1.94	0.76	1.97	0.72	3
Eosinophils	47	*10^^^9/l	−0.56	0.52	−0.48	0.64	2
Gamma glutamyltransferase	41	U/l	1.47	0.62	1.59	0.74	3
Glucose	50	mMol/l	0.62	0.93	0.67	0.37	1
Haematocrit	50	L/l	−0.40	0.00	−0.33	**0.01**	1
Haemoglobin	50	g/l	2.10	0.24	2.13	0.23	1
Indirect bilirubin	30	mMol/l	0.54	0.23	0.78	0.20	4
Iron	13	mMol/l	1.25	0.92	1.26	0.94	2
Lactate dehydrogenase	40	U/l	2.57	0.18	2.26	0.20	1
Lipase	23	U/l	0.83	0.20	1.56	0.06	3
Lymphocytes	50	*10^^^9/l	0.43	0.87	0.45	0.85	3
Magnesium	15	mMol/l	−0.16	0.60	−0.07	0.49	2
MCH	48	pg/cell	1.41	0.07	1.48	0.08	2
MCHC	50	g/l	2.51	0.00	2.54	**<0.01**	4
Mcv	48	fL	1.90	0.09	1.95	0.12	2
Monocytes	50	*10^^^9/l	−0.40	0.02	−0.07	**0.02**	5
Neutrophilic bands	31	*10^^^9/l	−0.52	0.00	−1.46	**<0.01**	2
Osmolarity	10	Osmol/l	−0.56	0.80	−0.56	0.92	2
Phosphorus	47	mMol/l	0.10	0.01	0.28	**0.01**	2
Platelet count	37	*10^^^12/l	−0.59	0.85	−0.57	0.84	4
Potassium	47	mMol/l	0.60	0.95	0.60	0.93	4
Red blood cell count	48	*10^^^12/l	0.70	0.24	0.65	0.27	1
Segmented neutrophils	50	*10^^^9/l	0.88	0.10	0.69	0.08	3
Sodium	49	mMol/l	2.14	0.34	2.15	0.38	2
Total bilirubin	49	μMol/l	0.76	0.35	0.93	0.31	2
Triglyceride	43	mMol/l	0.05	0.16	0.23	0.18	2
Uric acid	39	mMol/l	−0.96	0.17	−0.50	0.19	3
White blood cell count	50	*10^^^9/l	1.03	0.07	0.88	0.07	2

Probabilities in bold represent traits identified as significant given a false discovery rate of 10%. The final column provides the number of species similarly found to have values outside 95% of predictions made (including humans). All trait values are log_10_ transformed.

These analyses focused on change in single traits. To test the overall hypothesis that humans are recognized as an outlier for more physiological traits than other primate species—i.e., that humans are ‘generally’ different from other primates—we ran the same BayesModelS analysis for every primate species for each trait. This provided information on the number of outlier traits for each species on the tree, while also helping to assess the validity of our phylogenetic-statistical model. We then ran BayesModelS a final time using the logit-transformed proportion of outlier traits for each species as the test variable, and including the number of traits for which each species was included as a predictor variable. We predicted the proportion of outlier traits in humans and tested whether humans departed from this prediction.

The statistical performance of BayesModelS has been tested in general (12), but not in the specific case of changes along the human lineage. Although not expected, it is possible that some topologies would tend to lead to an excess of outliers. To ensure that our inference of human outliers is not an artifact of the tree topology, we created R code to simulate evolution along with the tree, with either no excess change along the human lineage (thus testing for the rate of false positives), or with different levels of excess change (testing for the rate of false negatives). We generated 100 datasets by simulating trait evolution under Brownian motion on our consensus primate tree, and an additional 150 datasets generated by simulating with a rate multiplier on the human branch, with this rate varying continuously from one to four. We then used BayesModelS to find the proportion of datasets for which the simulated human trait was identified as an outlier. To enable others to use this code for their own datasets (including for any lineage on any phylogeny), we provide it in the Electronic Supplementary Material.

### Inferring regime shifts

We analysed a multi-state Ornstein-Uhlenbeck (OU) model with the bayou package (27) in R. Bayou uses a reversible-jump MCMC similar to the one used in BayesModelS, but instead of generating a linear regression equation, bayou estimates the parameters of an OU model of evolution and calculates the likelihood of the data given the model. As a fully Bayesian implementation of the OU model, bayou overcomes many concerns of biased estimates when modelling OU evolution (28). Bayou estimates values for trait optimum (Θ), strength of mean reversion to each optimum (α), the rate of evolutionary change (σ^2^), the number of changes in Θ across the phylogeny (k), and where changes in Θ occur on the phylogenetic tree. For comparison to the BayesModelS analyses, we used a developmental version of bayou that included a predictor variable (body mass). However, bayou will only accept a single tree for analysis, and is thus unable to account for phylogenetic uncertainty. All prior distributions were created using the function make.prior in the R package “bayou,” with the model type set to “ffancova” which provides values to an assigned distribution type for each parameter based on the data and the tree. A half-Cauchy distribution was assigned to α and σ^2^, a uniform distribution to the regression coefficient of log mass (β), a Poisson distribution to k, a normal distribution with mean and standard deviation equivalent to the distribution of values for the trait being tested was assigned to Θ, and a uniform distribution was assigned to the probability that a change in Θ occurs on a given branch, with the restriction that only one change can occur on any branch. Two MCMCs were run for 2,002 000 steps, with the first 2000 steps discarded as burn-in, and thin rate of every 1000 steps thereafter (resulting in MCMC chains with 2000 samples). After discarding initial burn-in, Gelman’s R statistic was used to evaluate the convergence of the two chains run for each trait. An additional proportion of each chain was discarded to bring Gelman’s R for the likelihood of models across both chains as close as possible to one. The two chains were then combined for all additional analyses, and effective sample sizes were estimated based on the log likelihoods of the remaining models. We analysed the output from bayou to obtain the proportion of models that inferred a regime shift on the human lineage. We also obtained the magnitude of that change relative to other changes on the tree. To operationalize whether humans are recognized as experiencing a change in selective regime, we required that at least 20% of models inferred a shift on the human tip and at least 95% of those changes had to be consistently in one direction.

## RESULTS

### Phylogenetic prediction

**Figure 1.**
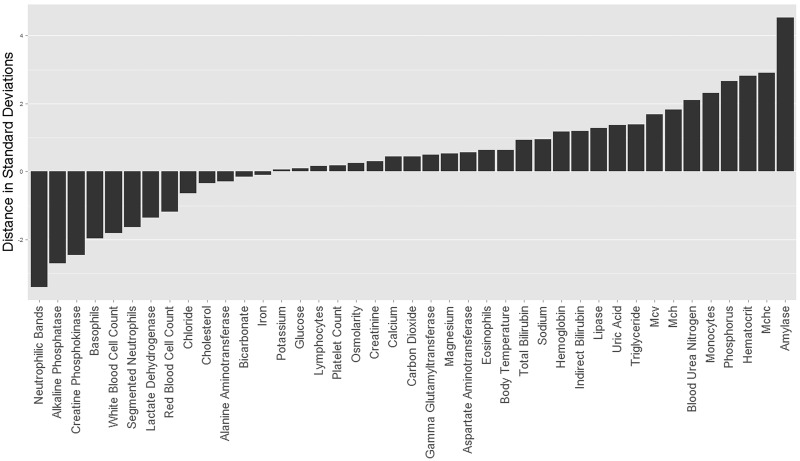
Results from phylogenetic prediction using BayesModelS. Bars show the distance in standard deviations of the actual human value for each trait from the mean of predictions in the posterior distribution generated for that trait by BayesModelS

Human values for monocytes, phosphorous, haematocrit, MCHC and amylase all received substantial support as higher than predicted. Human values for creatine phosphokinase, alkaline phosphatase and neutrophilic bands were all substantially lower than predicted. Thus, human values for eight traits were determined to fall outside the range expected by our phylogenetic predictions (Fig. 1). Some traits associated with aerobic function (haematocrit, MCHC) or immunocompetence (monocytes) are amongst the traits identified as evolutionary outliers, as predicted. Specific probabilities for all traits are presented in Table 2.

**Figure 2.**
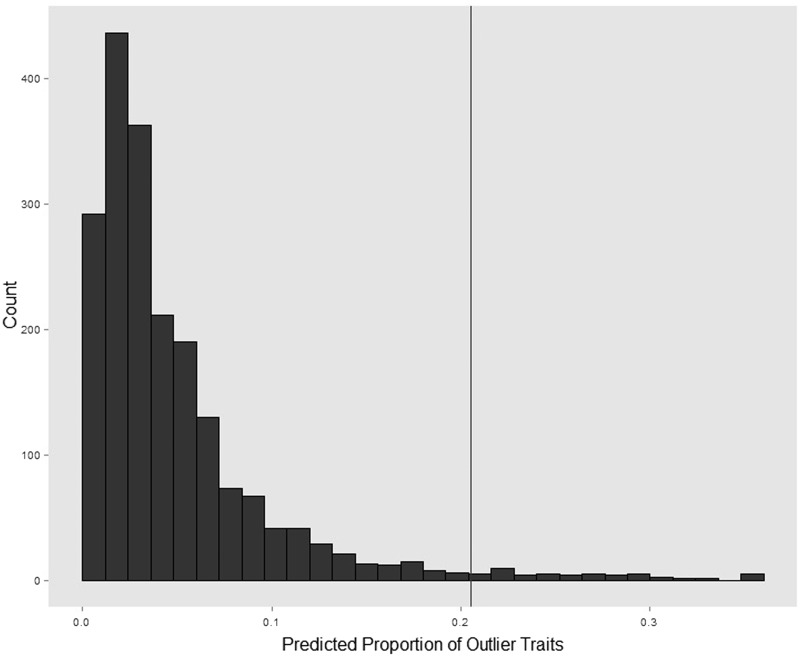
Humans have more traits identified as outliers. Posterior distribution of proportion of human traits predicted to be outliers using BayesModelS. Vertical line indicates the observed value for humans

We also investigated whether other primates in the dataset have traits with unexpected values. These analyses revealed that humans are outliers for more traits than any other primate. On average, non-human primate species had an unexpected high or low value for 6.4% of traits tested (SD = 4.9%), compared to 20.5% of traits for humans. When predicting the proportion of outlier traits in humans, only 2.6% of predictions were larger than the actual value of 20.5% (Fig. 2). Tree topology also does not appear to have played a role in the high proportion of human outlier traits. Only 5% of simulations produced outliers on the human tip when using a constant rate Brownian motion model across the tree including all primates used across our analyses. When we simulated data using Brownian motion with a rate multiplier on the human tip, the percentage of simulations with human outliers reached 20% when the rate ranged from 3 to 4 (Supplementary Material, Fig. 1), suggesting that the method requires large changes for detection of outliers on the human lineage, at least under Brownian motion. Thus, it appears that humans show a general pattern of altered physiology, with an unexpected number of outlying physiological traits.

### Modelling regime shifts using an Ornstein-Ulenbeck analysis

Inspection of MCMCs revealed moderate auto-correlation of models in the posterior distributions. All chains reached convergence, indicating that our parameters were estimable and reducing concerns about over-parameterization of the OU model raised by Ho & Ané (28). Large overall chain size resulted in acceptable effective sample sizes for model likelihood (between 200 and 4000) for all traits except potassium, glucose and body temperature (Table 3). In addition, four traits (bicarbonate, iron, magnesium and osmolarity) did not have four times more species in the analysis than estimated regime shifts, a condition that resulted in reduced accuracy of estimates in previous simulations (27). Thus, we interpret results for these traits with caution.

**Table 3.** Summary of posterior distributions of models generated with bayou.

Trait	# Species	Eff. Sample Size (lnL)	Mean Θ shifts per model	Prop. models with Θ shift on human lineage	Branches in tree with more Θ shifts than human lineage	Mean Θ shift	SD Θ shift	Prop. Shifts on human lineage > 0
Alanine aminotransferase	50	1791	6.6	0.02	>10	-0.09	0.15	0.16
Alkaline phosphatase	50	3292	5.7	0.03	>10	-0.20	0.26	0.22
Amylase	35	863	5.9	**0.52**	0	0.93	0.32	**0.99**
Aspartate aminotransferase	50	1303	6.2	0.01	>10	0.18	0.26	0.82
Basophils	37	3115	6.1	0.06	>10	-0.13	0.19	0.21
Bicarbonate	16	3201	5.1	0.09	>10	0.00	0.07	0.53
Blood urea nitrogen	50	589	7.2	**0.20**	4	0.24	0.11	**0.99**
Body temperature	44	173	5.6	0.02	>10	0.01	0.00	**1.00**
Calcium	50	260	7.3	0.02	>10	0.01	0.02	0.78
Carbon dioxide	32	1901	6.0	0.13	2	0.05	0.03	**0.95**
Chloride	48	470	6.3	0.02	>10	-0.01	0.01	0.26
Cholesterol	49	774	7.8	0.04	>10	-0.03	0.14	0.48
Creatine phosphokinase	40	3804	6.0	0.06	>10	-0.06	0.34	0.46
Creatinine	50	1697	5.4	0.02	>10	0.04	0.05	0.84
Eosinophils	47	1721	7.3	0.04	>10	0.07	0.13	0.74
Gamma glutamyltransferase	41	1845	6.0	0.09	5	0.34	0.27	0.91
Glucose	50	153	7.5	0.01	>10	0.06	0.11	0.79
Haematocrit	50	460	7.1	**0.48**	1	0.07	0.03	**0.99**
Haemoglobin	50	1562	5.9	0.04	>10	0.03	0.03	0.84
Indirect bilirubin	30	907	6.4	0.12	7	0.18	0.16	0.84
Iron	13	3013	5.4	0.12	>10	-0.03	0.13	0.38
Lactate dehydrogenase	40	3525	5.9	0.06	>10	-0.12	0.29	0.34
Lipase	23	2446	5.6	**0.49**	0	0.63	0.28	**0.98**
Lymphocytes	50	1624	6.2	0.03	>10	0.01	0.11	0.57
Magnesium	15	1637	6.1	**0.21**	6	0.09	0.13	0.82
Mch	48	330	7.2	0.09	>10	0.05	0.09	0.89
Mchc	50	3315	5.9	0.06	3	0.01	0.01	0.77
Mcv	48	621	6.9	0.08	>10	0.04	0.09	0.90
Monocytes	50	921	6.2	**0.60**	2	0.29	0.15	**1.00**
Neutrophilic bands	31	3265	5.7	**0.27**	4	-0.47	0.03	**0.03**
Osmolarity	10	264	6.2	**0.20**	>10	-0.01	0.03	0.43
Phosphorus	47	1109	7.1	0.19	3	0.15	0.10	**0.98**
Platelet count	37	1406	6.4	0.03	>10	0.02	0.12	0.57
Potassium	47	30	7.9	0.03	>10	0.00	0.06	0.37
Red blood cell count	48	453	7.2	0.03	>10	-0.03	0.08	0.21
Segmented neutrophils	50	320	7.2	0.02	>10	0.01	0.14	0.58
Sodium	49	381	6.3	0.02	>10	0.00	0.01	0.47
Total bilirubin	49	2421	5.9	0.05	>10	0.10	0.16	0.79
Triglyceride	43	2600	6.2	0.11	>10	0.15	0.13	0.91
Uric acid	39	216	9.5	0.06	>10	0.41	0.56	0.93
White blood cell count	50	974	6.3	0.03	>10	-0.04	0.09	0.28

For each trait, the effective sample size of the likelihood (natural log) of models in the MCMC, the mean and standard deviation of the number of Θ shifts per model in the posterior distribution, the proportion of models with an optimum shift on the *Homo* tip, the rank of the *Homo* tip amongst all branches in the tree in terms most optimum shifts on the given branch, the mean and standard deviation of all shifts on the *Homo* tip, and the proportion of those shifts that are positive. Optimum shift values represent log_10_ transformed data.

For all traits, the average number of adaptive regime changes that occurred on the tree ranged from five to eight, with the exception of uric acid, which averaged 9.53 regime changes per model (Table 3). We used several measures to estimate the magnitude of evolutionary change since the humans’ common ancestor with *Pan* (Table 3). Across traits, the average percentage of models in the posterior distribution with an optimum change on the branch to *Homo* is 11.7% (SD = 15.0%). Several traits contain an adaptive regime shift on the *Homo* branch at high frequencies: monocytes (60.3%), amylase (52.1%), lipase (48.8%) and haematocrit (48.2%). For amylase and lipase, the *Homo* branch contains an optimum change in a greater proportion of models than any other branch in the primate tree; for monocytes, haematocrit, neutrophilic bands, blood urea nitrogen, phosphorus, carbon dioxide and MCHC, the *Homo* branch is in the top five branches.

**Figure 3.**
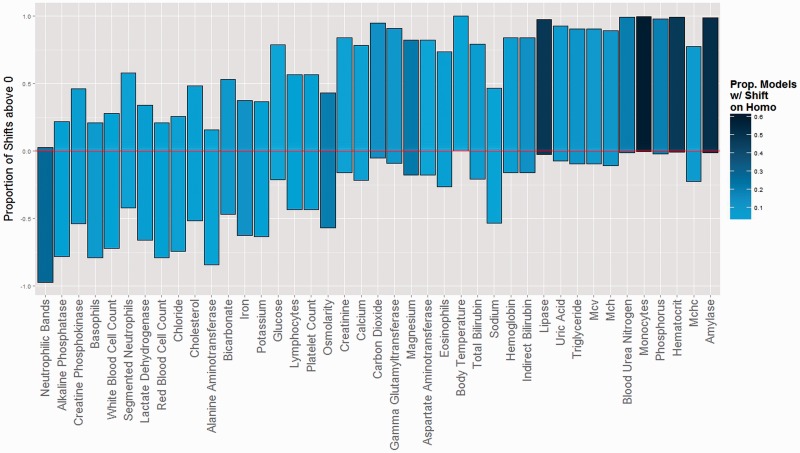
Results of modelling adaptive regimes in the OU model. Each bar represents the proportion of adaptive regime shifts occurring on the *Homo* tip that are positive (top of bar) and negative (bottom of bar) for each trait. Shading represents the proportion of models in the posterior distribution for which a Θ shift occurs on the *Homo* tip, where darker shades mean a higher proportion of models. Bars that are almost entirely above or below zero (red line) and have a darker fill indicate stronger evidence of an evolutionary change in recent human evolution. Traits are listed on the x-axis in the same order as Fig. 1 for comparison

In addition to identifying regime shifts, it is crucial to assess whether the optimum (Θ) shift direction is consistently in one direction. The proportion of models that contain a Θ shift of the *Homo* tip and the directionality of the shifts are visualized simultaneously in Fig. 3. When considering only models for which the *Homo* tip contains a regime change, 95% or more of the Θ shifts are positive for body temperature, blood urea nitrogen, amylase, phosphorus, lipase and carbon dioxide. Consistent with *a priori* predictions, we also found positive shifts along the human lineage for monocytes and haematocrit. For neutrophilic bands, 95% of Θ shifts on the *Homo* tip are below zero.

## CONCLUSIONS AND IMPLICATIONS

The approaches that we used represent new ways to examine evolution on a single branch, based on variation across species in the clade of interest and phylogenetic-statistical modelling. The specific methods expand on previous approaches (10,11) by employing Bayesian MCMC to incorporate uncertainty in estimated parameters and phylogeny, and by explicitly modelling evolutionary change along single branches (the OU model) or by making phylogenetically informed predictions for humans (phylogenetic prediction using PGLS). In the latter case, the analysis is essentially predicting the phenotype of humans as if we were ‘typical’ primates, given covariation between the physiological values and body mass. Thus, any deviations from predictions suggest that humans have undergone substantial evolutionary change based on predictor variables that were not incorporated in the model for understanding other primates, and potentially involving unique selective pressures. These predictions also incorporate phylogeny when making the prediction, based on the degree of phylogenetic signal in the statistical models.

Focusing on the traits supported in both analyses, we find that the following five traits reached our criteria for undergoing substantial evolution along the human lineage: amylase, haematocrit, phosphorus, monocytes and neutrophilic bands. Some additional traits were supported as outliers in one analysis, but not the other, including MCHC, creatine phosphokinase, alkaline phosphatase and lipase. More generally, we found a striking pattern that humans have more traits identified as outliers in the phylogenetic prediction model than other primates (where twelve species were identified as having no exceptional traits). This pattern suggests that broad physiological changes are more common in recent human evolution than for any other primate analysed. Alternatively, the large number of outlier traits for humans may be attributable to obtaining our human data from a different source than the other primates, and may reflect effects of captivity on physiology in non-human primates.

For some of the traits, we were able to formulate specific *a priori* predictions for differences in humans. One hypothesis involved the importance of endurance running in human evolution (15). Under this hypothesis, we predicted that variables related to oxygen transport in the blood would show higher values in humans, including haematocrit, red blood cell counts, haemoglobin and MCHC. We found strong support for an increase in haematocrit over the human lineage in both analyses, although red blood cells and haemoglobin were not among the traits identified as evolutionary outliers in humans.

We also predicted increases in white blood cells along the human lineage, based on hypotheses that humans have undergone multiple epidemiological transitions (29). Specifically, several factors in human evolution may have facilitated an over-abundance of parasites and pathogens, including more sedentary lifestyles, close affiliation with domesticated animals and their parasites and pathogens, and contact with rodents and other animals that pilfer stored food items (16,29). We found support for one specific type of white blood cell—monocytes—to show increases along the human lineage, although other cell types (and overall counts) did not show significant increases (see also ref. 12). We also found strong evidence for a decrease in neutrophilic bands (a type of immature white blood cell) in the human lineage. It remains unclear why other cell types, such as mature neutrophils, eosinophils and lymphocytes, do not show corresponding increases along the human lineage, or why neutrophilic bands decreased. We thus draw cautious conclusions concerning the role that pathogen load has had on the evolution of human physiology. Further research could investigate more complex models that account for additional ecological and social variables, although this was not found to improve predictive capacity for leukocyte counts in a previous BayesModelS analysis (12).

Amylase was also among the best supported of the traits that we investigated. In hindsight, this makes sense in relation to changes in the human diet, although it was not among our *a priori* hypotheses. In particular, previous work has suggested that the number of copies of the amylase gene increased in humans; these increases are proposed to relate to increased consumption of starch in the human diet (30). Our findings suggest that the increased copy number has physiological outcomes that show up in rigorous evolutionary modelling along the human branch.

The findings from our study may provide insights into human diseases, and even novel solutions for controlling disease. We propose that traits undergoing rapid change—and in relation to potentially novel selective forces—may generate new tradeoffs with other traits, for example, through antagonistic pleiotropy (1) and evolutionary inertia (or ‘evolutionary lag’, ref. 31). A major concept in applications of the OU model is that traits may exhibit inertia, which can be estimated (32). Results are mixed as to which traits show inertia and the reasons for this, with several studies of primates failing to find compelling evidence for evolutionary lag (31,33,34). An improved understanding of how traits respond to changes in other traits may help to uncover constraints that limit counter-measures that would offset the costs of rapidly evolving traits. This may be especially true for physiological parameters, which can be both costly to produce (e.g., immune system cells) or detrimental to health through effects on other body systems (e.g., cholesterol, testosterone).

One cautionary note in interpreting our data concerns our use of human values from Western countries where hygiene, access to healthcare and sedentary lifestyles may affect the physiological values. Some changes in physiological values may be a response to behavioural changes, rather than traits shaped by natural selection, often with non-intuitive effects. For example, fitness levels may mediate traits like haemoglobin; in some studies, trained athletes have shown lower levels of haematocrit, haemoglobin concentration and total blood volume than other individuals from the same population (e.g., 35), and higher levels of these traits in other studies (e.g., 36). Similarly, Blackwell *et al.* (37) studied immune traits of an Amazonian population in a parasite rich environment and found elevated levels overall, with particularly high values of eosinophils. Although parasite load itself may explain this pattern, these results highlight the need for more comprehensive human data and complicate the lack of difference we found in many immune traits, including eosinophils. Alternatively, one could argue that eosinophil counts in those without helminth infections—such as our use of data from Western populations—is more relevant to assessing evolutionary variation in baseline defences, provided of course that the other primates in the sample experience similar conditions (which, as zoo animals with access to medicine and veterinary care, they do). Further studies comparing different human populations could elucidate the interactions between evolutionary changes and phenotypic plasticity that we are unable to address with our current data set.

While some of the traits we predicted to be outliers in our *a priori* hypothesis did indeed show a strong effect, some traits that were predicted to show marked change failed to do so. For example, our results revealed support for one type of white blood cell, but not for increased overall white blood cell count. Similarly, we found support for increased haematocrit along the human lineage, but not for overall red blood cell counts. This suggests that adaptations in traits for long distance running and increased pathogen load were either too small to detect, or occurred only in specific aspects of human physiology. In the latter case, changes like those we found for monocytes or haematocrit may be revealing, but should be investigated further. Another caveat concerns the consistency of the association between the physiological reference values and disease across species. For example, we do not know whether similar values of haematocrit reflect similar levels of aerobic fitness in humans, other apes and monkeys. Future studies may use methods similar to ours, but also include additional predictor variables and intraspecific variation, optimize prior distributions for an individual trait, or explore the interactions of changes in multiple physiological values. Comparative analyses may also be conducted among different human populations thought to differ in selective pressures on these phenotypes.

The methods we apply here could be used to study the evolution of other traits that are relevant to human health. Recently, for example, Samson and Nunn (13) investigated human sleep in evolutionary context, finding that humans sleep much less than one would predict for a primate with our body mass, brain size, diet, activity pattern and life history traits. They further showed that humans spend a greater proportion of the night in rapid eye movement sleep than expected. This approach could be applied to additional traits, such as those associated with bipedal locomotion, childbirth, or brain size. Many of these gross changes in human evolution are likely linked to disease or health consequences, such as difficulty in childbirth. Yet, few studies have quantitatively assessed the specific morphological features that have undergone rapid change along the human lineage.

In conclusion, we found that several physiological traits have undergone rapid and exceptional change along the human lineage in ways that differ from other primates, and that selective regimes have commonly changed along the human lineage. A critical step for future research will be to understand more about how these ‘exceptional’ traits change with other physiological, morphological and behavioural traits across primates, and how these may represent constraints or tradeoffs along the human lineage with consequences for understanding how evolution has made us susceptible to disease.

## Supplementary Material

Supplementary Data
