# Supplementary material for: Evolutionary change in physiological phenotypes along the human lineage
Source: Evol Med Public Health. 2016 Sep 11;2016(1):312–24. doi: 10.1093/emph/eow026 (PMC5046993; doi:10.1093/emph/eow026)
Supplement: Supplementary Data [file supp_2016_1_312__index.html]

Evolutionary Change in Physiological Phenotypes Along the Human Lineage — Evolutionary change in physiological phenotypes along the human lineage — Supplementary Data 

# Evolutionary change in physiological phenotypes along the human lineage

## Supplementary Data

files

- Supplementary Data - jpeg file
- Supplementary Data - txt file
