# Supplementary figures and images for: Evolutionary change in physiological phenotypes along the human lineage
Source: Evol Med Public Health. 2016 Sep 11;2016(1):312–24. doi: 10.1093/emph/eow026 (PMC5046993; doi:10.1093/emph/eow026)

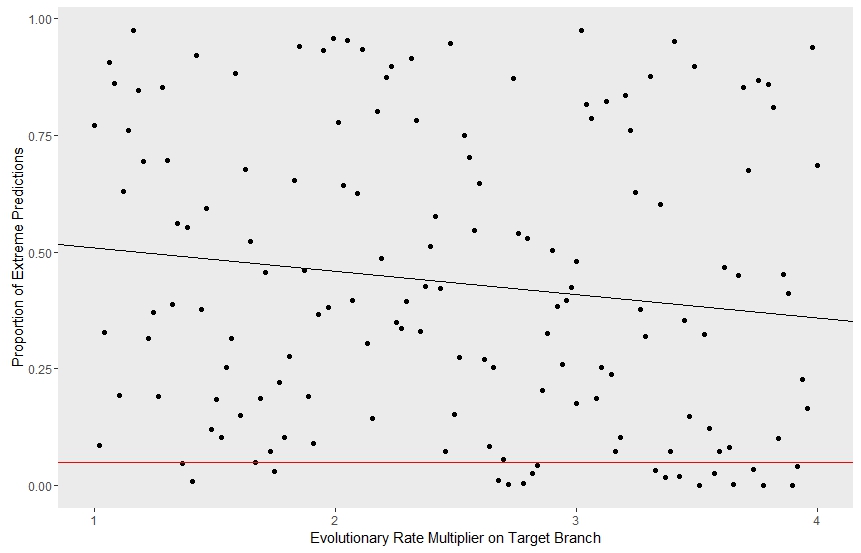

Supplement: Supplementary Data [file supp_eow026_Outliers_on_Human_Tip.jpg]
